# Targeting mitochondrial metabolism for precision medicine in cancer

**DOI:** 10.1038/s41418-022-01022-y

**Published:** 2022-07-13

**Authors:** Lourdes Sainero-Alcolado, Judit Liaño-Pons, María Victoria Ruiz-Pérez, Marie Arsenian-Henriksson

**Affiliations:** Department of Microbiology, Tumor and Cell Biology (MTC), Biomedicum B7, Karolinska Institutet, SE-171 65 Stockholm, Sweden

**Keywords:** Cancer metabolism, Glycobiology

## Abstract

During decades, the research field of cancer metabolism was based on the Warburg effect, described almost one century ago. Lately, the key role of mitochondria in cancer development has been demonstrated. Many mitochondrial pathways including oxidative phosphorylation, fatty acid, glutamine, and one carbon metabolism are altered in tumors, due to mutations in oncogenes and tumor suppressor genes, as well as in metabolic enzymes. This results in metabolic reprogramming that sustains rapid cell proliferation and can lead to an increase in reactive oxygen species used by cancer cells to maintain pro-tumorigenic signaling pathways while avoiding cellular death. The knowledge acquired on the importance of mitochondrial cancer metabolism is now being translated into clinical practice. Detailed genomic, transcriptomic, and metabolomic analysis of tumors are necessary to develop more precise treatments. The successful use of drugs targeting metabolic mitochondrial enzymes has highlighted the potential for their use in precision medicine and many therapeutic candidates are in clinical trials. However, development of efficient personalized drugs has proved challenging and the combination with other strategies such as chemocytotoxic drugs, immunotherapy, and ketogenic or calorie restriction diets is likely necessary to boost their potential. In this review, we summarize the main mitochondrial features, metabolic pathways, and their alterations in different cancer types. We also present an overview of current inhibitors, highlight enzymes that are attractive targets, and discuss challenges with translation of these approaches into clinical practice. The role of mitochondria in cancer is indisputable and presents several attractive targets for both tailored and personalized cancer therapy.

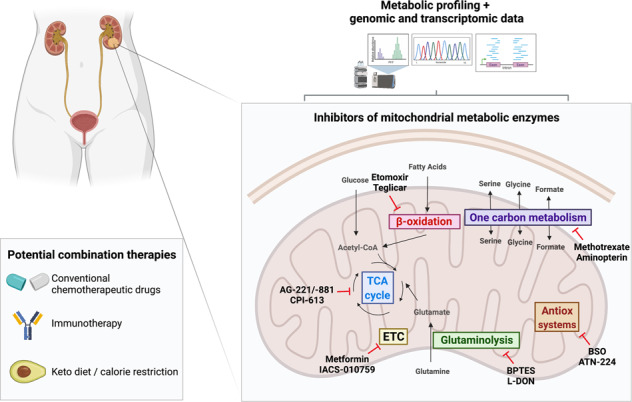

## Facts

- Mitochondrial metabolism is reprogrammed in cancer, providing attractive targets for therapy.
- Although targeting mitochondrial metabolism proves challenging, several inhibitors of key enzymes are currently in clinical trials.
- Combination strategies and novel drugs against metabolic pathways may provide a potential advantage for precision medicine in cancer.

## Open questions

- Why is it challenging to develop efficient inhibitors for mitochondrial metabolic enzymes?
- Which are the mitochondrial enzymes that need more specific and less toxic inhibitors?
- Will combination therapies, including immunotherapy/conventional chemotherapeutics/ketogenic or calorie restriction diets together with inhibition of mitochondrial metabolism improve the survival of cancer patients?

## Introduction: Tumor metabolism

Cancer cells are characterized by the ability to proliferate uncontrollably in contrast to normal cells, which are tightly regulated. To maintain rapid cell proliferation, tumor cells activate and/or modify metabolic pathways to obtain more energy, known as metabolic reprogramming. This research field has gained special interest during the latest decade due to new insights into its fundamental importance and the development of novel biochemical and molecular tools. In 2011, Douglas Hanahan and Robert A. Weinberg included metabolic reprogramming as one of the capabilities acquired during malignant transformation, i.e., one of the hallmarks of cancer [1].

In the 1920s, Otto Warburg described that tumor cells consume high amounts of glucose to produce lactate via “aerobic glycolysis” regardless of the presence of oxygen, generating only two molecules of ATP per molecule of glucose, versus the 36 ATP molecules produced during oxidative phosphorylation (OXPHOS) in normal cells [2, 3]. To overcome the 18-fold difference in efficiency, glycolysis is activated by upregulation of glucose transporters (including GLUT1) to increase glucose uptake and by overexpression of the rate-limiting enzyme in glycolysis, hexokinase-2, and lactate dehydrogenase (LDHA). The increase in glycolytic flux results in accumulation of glycolytic intermediates that supply different biosynthetic pathways to fulfill the demands of proliferation, e.g. the pentose phosphate pathway (PPP) for production of ribose and cytosolic nicotinamide adenine dinucleotide phosphate (NADPH) used for nucleotide synthesis and antioxidants; as well as one carbon metabolism necessary for mitochondrial NADPH production, methylation, and nucleotide synthesis [4].

The metabolic alterations in tumor cells are mainly driven by aberrant activation of the phosphatidylinositol-3 kinase-protein kinase B-mammalian target of rapamycin (PI3K-AKT-mTOR) pathway and activation of oncogenes such as *MYC*, *RAS*, and *hypoxia-inducible factor 1* (*HIF-1*), as well as mutations or deactivation of tumor suppressor genes including *p53* and *PTEN* (*phosphatase and tensin homolog*) (Supplementary Box 1).

### The Warburg effect and the relevance of OXPHOS

Warburg hypothesized in 1956 that tumor cells rely more on aerobic glycolysis due to an injury in respiration, which led to the misconception that cancer cells have a defective oxidative metabolism. However, Warburg’s experiments indicated that tumors consume oxygen at a lower rate compared to their increased glucose consumption [3]. Several studies have demonstrated that many cancer cells have the capacity to oxidize glucose via OXPHOS in their fully functional mitochondria (Supplementary Table 1). Moreover, inhibition of glycolysis does not prevent tumorigenesis. For instance, inhibition of the pyruvate kinase M2 isoform, responsible for the last step of glycolysis, still results in tumor formation in a breast cancer model [5]. Furthermore, inhibiting LDHA increases mitochondrial respiration in mammary tumor cells, proving that oxidative metabolism still is functional [6]. Therefore, cancer cells may depend equally or predominantly on OXPHOS for ATP supply, with the exemption of tumors with mutations in the tricarboxylic acid (TCA) cycle enzyme genes *SDH*, *IDH*, and *FH*, important during mitochondrial respiration. However, tumors carrying such mutations are still dependent on mitochondrial activity, and are remodeling their metabolism to optimize production of reactive oxygen species (ROS) and produce TCA cycle intermediates necessary for cell proliferation [7, 8].

## Mitochondria

Mtochondria are the organelles responsible for energy production in cells. The discovery of the mitochondrial genome (mtDNA) and phylogenetic gene analysis showed that mitochondria are descendants of an endosymbiotic α-proteobacteria (Supplementary Box 2). They consist of an outer (OMM) and an inner membrane (IMM), limiting the intermembrane mitochondrial space, and an electron-dense matrix. The IMM folds in the matrix forming the *cristae*, which contain the mitochondria respiratory machinery and whose density varies depending on the energy demand. The OMM is permeable, allowing diffusion of molecules up to 5 kDalton via the porin voltage dependent anion channel, receiving signals that will be transmitted into mitochondria [9]. It also serves as a contact site for interaction with other organelles including the endoplasmic reticulum, lysosomes, peroxisomes, endosomes, the plasma membrane, and lipid droplets [10].

Mitochondria are highly dynamic and stay in constant turnover through mitogenesis, mitophagy, and fusion–fission processes (Supplementary Box 3 and Supplementary Fig. 1). They control a vast number of cellular processes related to metabolism, apoptosis, redox homeostasis, calcium signaling, and iron metabolism, thus their integrity is fundamental for the cell (Supplementary Box 4).

### Mitochondrial metabolism

Mitochondria are crucial bioenergetic hubs. Virtually all metabolic fuels can be entirely oxidized to CO_2_, water, and ATP through the convergence into acetyl coenzyme A (acetyl-CoA), and its funneling into catabolic processes. The major mitochondrial metabolic pathways are the TCA cycle, fatty acid oxidation (FAO), the electron transport chain (ETC), and OXPHOS, all involved in catabolism of biomolecules and energy production. In addition, mitochondria provide precursors for many biomolecules and adapt to different metabolic conditions by modifying nuclear transcription [11]. All these processes have potential roles in development and maintenance of malignant phenotypes and understanding how they function in different cancer types may provide insights for new therapeutic approaches.

## Inhibitors of mitochondrial metabolic enzymes in precision medicine of cancer

Cancer represents the second leading cause of deaths worldwide. The main therapeutic approaches include surgery, chemotherapy, and radiation. However, the lack of specifity of chemotherapeutic agents translates into systemic toxicity and, with time, into relapse. Moreover, the heterogeneity of cancer is also associated with chemotherapy resistance, challenging effective treatment.

Immunotherapy has emerged as a promising approach to target cancer, including checkpoint blockage, adoptive T cell transfer, and cancer vaccines. The work of James P. Allison and Tasuku Honjo on cytotoxic T-lymphocyte-associated antigen 4 (CTLA-4) and programmed cell death protein 1 (PD-1) for cancer therapy was awarded with the Nobel Prize in Physiology or Medicine in 2018 (www.nobelprize.org). Although immunotherapy is proven to be more successful than the most effective chemotherapeutic drugs, it can also cause severe side effects.

The increased knowledge on the deregulation of cancer metabolism has set focus on precision medicine for targeting of each individual tumor type. However, there are several reasons why metabolism-targeted therapy is challenging. First, it is difficult to develop specific inhibitors as many metabolic enzymes have several isoforms with high structural similarity, e.g., glutaminase 1 and 2 [12] as well as pyruvate kinase 1 and 2 [13]. Second, many of these enzymes have hydrophobic pockets in their active sites, which are difficult to target [14]. Third, cancer cells can reprogram their metabolism, and thus, blocking one pathway by targeting a key enzyme could result in activation of another metabolic hub [15]. Fourth, inhibition of metabolism may cause adverse effects in normal cells.

Even though there are difficulties to identify specific compounds targeting metabolic enzymes, several pro-drugs are currently in clinical trials for treatment of both solid and hemaetological tumors. In the following sections we discuss the importance of mitochondrial metabolism in cancer and available inhibitors for each pathway, highlighting the need for more specific compounds for translation to clinical use.

## Mitochondrial metabolic pathways in cancer development

### The tricarboxylic acid (TCA) cycle

**Fig. 1:**
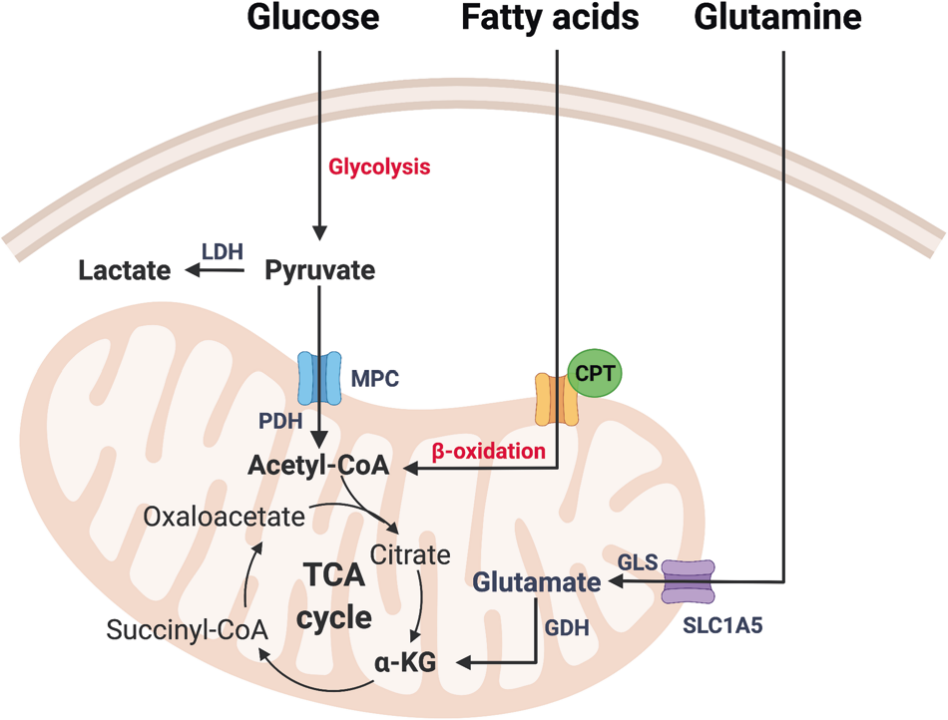
Tricarboxylic acid (TCA) cycle fuels. Carbons derived from glucose, fatty acids, and glutamine enter the TCA cycle, which produces NADH and FADH_2_, necessary for the formation of ATP through the electron transport chain (ETC). Glucose is processed into pyruvate via glycolysis, resulting in lactate by lactate dehydrogenase (LDH) or enters the mitochondria through the mitochondrial pyruvate carrier (MPC). Once in the mitochondria, pyruvate will be converted into acetyl-CoA by pyruvate dehydrogenase (PDH). Fatty acids are transferred to the mitochondria by carnitine palmitoyltransferases (CPT1/2) and provide acetyl-CoA to the TCA cycle through β-oxidation. Glutamine enters mitochondria through the solute carrier family 1 member 5 (SCL1A5) transporter and is converted into glutamate by glutaminases (GLS) and into α-ketoglutarate (α-KG) by glutamate dehydrogenase (GDH).

The tricarboxylic acid (TCA) cycle, also named Krebs cycle or citric acid cycle, is a central hub for generation of energy and macromolecules for biosynthetic purposes, as well as for redox balance. The TCA cycle reactions occur in the mitochondrial matrix, allowing oxidation of different fuels (Fig. 1). Carbons derived from glucose, fatty acids, or glutamine, enter the TCA cycle and produce NADH and flavine adenine dinucleotide (FADH_2_), which in turn transfer electrons to the ETC in the IMM, generating CO_2_ and ATP. The multiple substrates that supply the TCA cycle together with metabolic reprogramming allow cancer cells to survive in different situations of energy demand. This is dictated by a combination of mutations in oncogenes, tumor suppressor genes, as well as in the tumor microenvironment (TME) [1]. In hypoxia, most cancer cells convert pyruvate into lactate, fueling the TCA cycle with glutamine [16].

Furthermore, the TCA cycle provides intermediates for the production of lipids, proteins, and nucleotides. These need to be replaced to keep the TCA cycle functioning, a process called anaplerosis. Two anaplerotic pathways are glutaminolysis [17], producing α-ketoglutarate from glutamine, and pyruvate carboxylation, generating oxalacetate from glucose derived pyruvate [18]. Both energy production and biosynthetic pathways need to converge to maintain the pool of metabolic intermediates during low or high energy consumption, i.e., amid fasting or exercise, respectively.

#### Mutations in TCA cycle enzymes in cancer

Mutations in genes encoding enzymes of the TCA cycle have been associated with cancer progression, especially succinate dehydrogenase (*SDH*), fumarate hydratase (*FH*), and isocitrate dehydrogenase (*IDH*) (Supplementary Table 2). Moreover, these mutations cause abnormal accumulation of different metabolites, so called oncometabolites, resulting in deregulation of signaling promoting cancer progression.

Succinate dehydrogenase (SDH) is a hetero-tetrameric protein, formed by the catalytic subunits SDHA, SDHB, and the ubiquinone-binding and membrane-anchorage subunits SDHC and SDHD, which catalyze oxidation from succinate to fumarate. In addition, it participates as part of Complex II of the ETC, reducing ubiquinone to ubiquinol. Mutations in SDH subunits and in the SDH assembly factor 2 have been correlated with hereditary paraganglioma, pheochromocytoma [19, 20], gastrointestinal stromal tumors, renal cell carcinoma (RCC), thyroid tumors, testicular seminoma, and neuroblastoma [21–25].

Fumarate hydratase (FH) is responsible for the hydration of fumarate to malate. Patients harboring *FH* mutations have predisposition to develop multiple cutaneous and uterine leiomyomas, hereditary leiomyomatosis as well as renal cell cancer [26], and they may also be associated with ovarian and leydig cell tumors [27]. Mutations in *SDH* and *FH* result in the accumulation of succinate and fumarate, respectively. Both *SDH* and *FH* are considered tumor suppressor genes [28].

Isocitrate dehydrogenase (IDH), which comes in three isoforms, IDH1, IDH2, and IDH3, catalyzes the oxidative carboxylation of isocitrate to produce α-ketoglutarate (α-KG). Different cancers have mutations in *IDH1* and *IDH2*, including a fraction of acute myeloid leukemia, low-grade glioma, secondary glioblastoma, chondrosarcoma, and cholangiocarcinoma [29–32]. Contrary to *FH* and *SDH*, *IDH* mutations result in gain of function, converting α-KG to the oncometabolite 2-hydroxyglutarate (2-HG). Additional information regarding mutations in other TCA cycle enzymes as well as oncometabolites is presented in Supplementary Boxes 5 and 6.

#### Inhibitors of TCA cycle enzymes

**Fig. 2:**
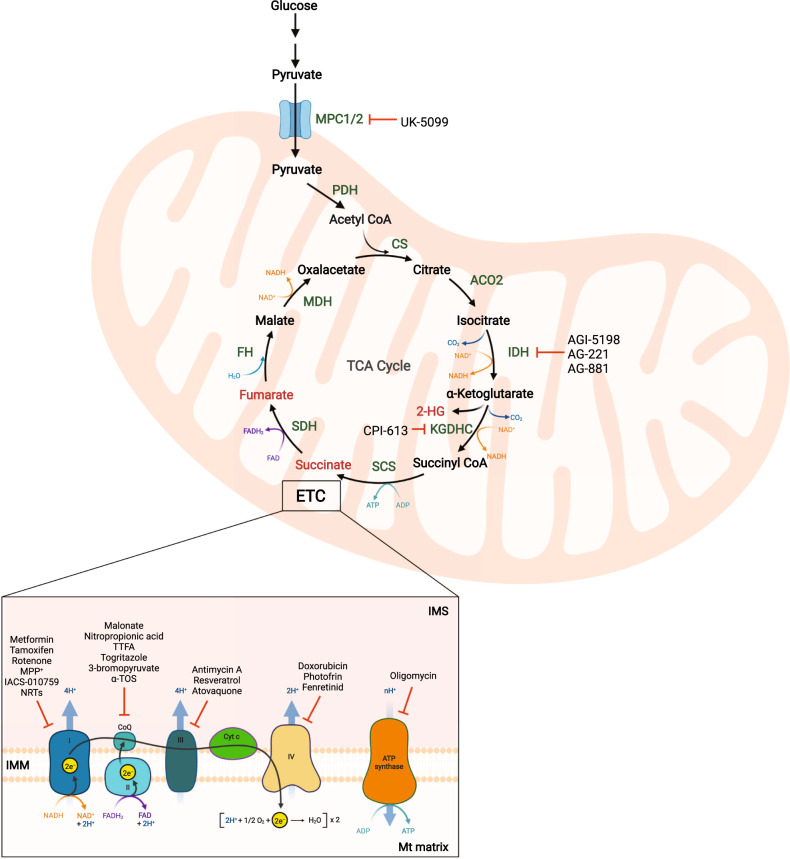
Targets of the tricarboxylic acid cycle (TCA) cycle and the electron transport chain (ETC). Glucose-derived pyruvate enters mitochondria via MPC1/2 transporters and is converted to acetyl-CoA via PDH. Inhibition of mitochondria pyruvate transporter using UK5099 attenuates OXPHOS. Alterations in enzymes of the TCA cycle results in production of oncometabolites. These include 2-HG, fumarate, and succinate (dark red). Inhibitors of these enzymes include AGI-5198, AG-221, and AG-881 for IDH, and CPI-613 for KGDHC. Both NADH and FADH_2_ produced in the TCA cycle transfer electrons to Complex I and II. Electrons then pass through a series of redox reactions producing energy for transportation of protons to the IMS, generating enough energy to produce ATP. Abbreviations: TCA tricarboxylic acid, ETC electron transport chain, MPC1/2 mitochondrial pyruvate carrier 1/2, PDH pyruvate dehydrogenase, CS citrate synthase, ACO2 aconitase 2, IDH isocitrate dehydrogenase, KGDHC α-ketoglutarate dehydrogenase complex, SCS succinyl-CoA synthetase, SDH succinate dehydrogenase, FH fumarate hydratase, MDH malate dehydrogenase, 2-HG 2-hydroxyglutarate, IMS intermembrane mitochondrial space, IMM inner mitochondrial membrane, Mt matrix mitochondrial matrix, FADH_2_ flavin adenine dinucleotide.

Several potential therapeutic strategies to target the TCA cycle for cancer treatment are summarized in Fig. 2. Although loss of function mutations of the FH or SDH enzymes are challenging to target, several small compounds have shown to be successful inhibitors for enzymes with gain of function, while others need improvement. Inhibition of mutant IDH by AGI-5198 reduces 2-HG formation and induces differentiation of glioma cells [33]. Notably, AG-221 [34] and AG-881 [35] are in clinical trials for treatment of acute myelogenous leukemia carrying *IDH2* or *IDH1/2* mutations, respectively. Similarly CPI-613, targeting both the α-KG dehydrogenase complex as well as pyruvate dehydrogenase, is in phase I/II clinical trials for leukemias, lymphomas, and small cell lung cancer (SCLC) [36, 37].

### The electron transport chain (ETC) and oxidative phosphorylation (OXPHOS)

The ETC, also known as the respiratory chain, consists of four complexes (CI-IV) bound to the IMM and two electron transfer carriers (ubiquinone and cytochrome c) [38, 39]. The redox reactions result in an electrochemical gradient that releases enough energy for ATP formation via OXPHOS. Both NADH and FADH_2_ are electron donors, while oxygen is the final electron acceptor. Protons are pumped from the mitochondrial matrix into the intermembrane space by complexes I, III, and IV. Next, Complex V (the ATP synthase) uses the proton motive force generated to deliver protons to the mitochondrial matrix as well as catalyzes the conversion of ADP + P_i_ to ATP [40] (Fig. 2 and Supplementary Box 7).

#### Mutations in the ETC

Mitochondrial DNA encodes thirteen subunits of complexes I, III, IV, and V. Several tumor types including colon and rectum adenocarcinoma, ovarian cancer, acute myeloid leukemia, and glioblastoma show somatic mtDNA mutations that affect Complex I (NADH dehydrogenase), III (cytochrome b), or IV (cytochrome c oxidase) [41]. Mitochondrial mutations have also been attributed to tumorigenesis in prostate cancer and hepatocellular carcinoma [42, 43]. Mutations in SDH (Complex II) result in ROS production and accumulation of succinate, which in turn inhibits HIF prolyl hydroxylase, thus activating the HIF pathway [28].

#### Inhibitors of the ETC

The ETC has long been exploited for therapy, with development of inhibitors for each complex, some of them approved as drugs for certain diseases (Fig. 2). Complex I can be inhibited by tamoxifen, increasing hydrogen peroxide production. This compound is used for treating pre-menopausal hormone-positive breast cancer [44]. Metformin also inhibiting Complex I, is approved for type-2 diabetes and is the most prescribed drug worldwide. Several clinical trials are conducted for its use in colorectal, breast, and prostate cancer [45, 46]. IACS-010759, a new promising inhibitor of Complex I, is in clinical trials for acute myeloid leukemia and certain solid tumors [47]. Rotenone and ﻿methyl-4-phenylpyridinium also inhibit Complex I, but are neurotoxic. However, deguelin, a rotenone analog, has potential as a chemotherapeutic drug [48]. There are several experimental inhibitors of Complex II, such as malonate, nitropropionic acid, thenoyltrifluoro-acetona, troglitazone, 3-bromopyruvate, and α-tocopheryl succinate, a vitamine E derivative [49]. To inhibit Complex III, antimycin A is used in experimental research while resveratrol has enrolled clinical trials for different types of cancer [50]. Atovaquone, approved for treatment of pneumocystis pneumonia and malaria, inhibits Complex III both in parasites and human cells and is in clinical trials in combination with chemotherapeutic drugs in non small cell lung cancer (NSCLC) [51]. Complex IV can be inhibited by doxorubicin, a DNA-intercalating chemotherapeutic drug, and the porphyrin photosensitizer photofrin, which is approved for esophageal cancer and NSCLC [52]. N-(4-Hydroxyphenyl) retinamide (fenretinid) is in clinical trials for different tumor types, including ovarian cancer, B-cell non-Hodgkin lymphoma, and breast cancer [53]. No promising inhibitors have been reported to date for Complex V, except for oligomycin, only suitable for experimental use [49]. Employing mitochondrial uncouplers is an alternative approach to impair ETC function. Compounds including niclosamide, nitazoxanide, oxyclozanide, FCCP/CCCP, BAM15, or SR4 short-circuit ATP synthesis by transporting protons across the IMM. Niclosamide is in phase I/II clinical trials for prostate and colon cancer, while nitazoxanide is in phase II for different forms of advanced cancers [54].

### One carbon metabolism

**Fig. 3:**
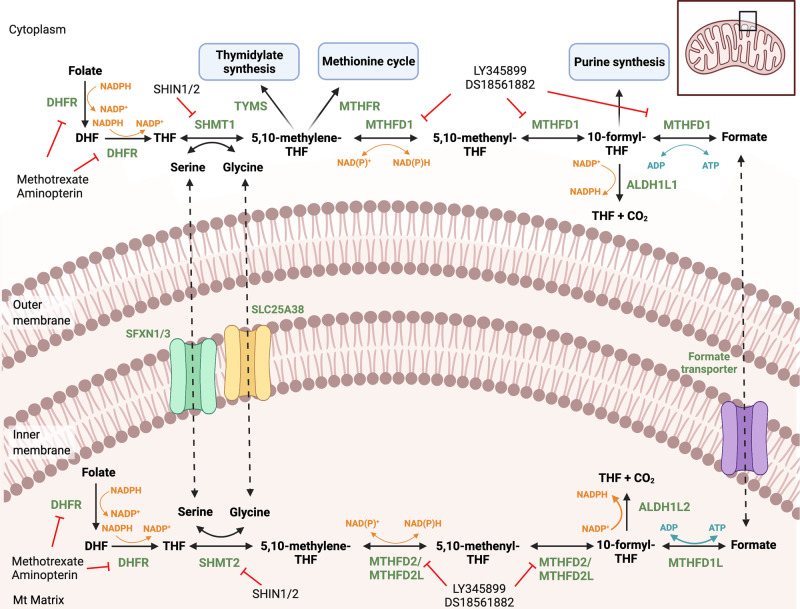
One carbon metabolism. Cytosolic and mitochondrial one carbon metabolism pathway indicating the available inhibitors for different enzymes. Folate is converted to DHF and further to THF via DHFR, a process that consumes NADPH. Serine is converted by SHMT1 (cytosolic) or SHMT2 (mitochondrial) to glycine. The one carbon unit resulting from the reaction is transferred to THF forming 5,10-methylene-THF, which is oxidized by cytosolic MTHFD1 or mitochondrial MTHFD2/MTHFD2L to 10-formyl-THF via the intermediate 5,10-methenyl-THF, generating NAD(P)H. The one carbon unit in 10-formyl-THF can be converted either to formate, generating ATP from ADP by MTHFD1 or MTHFD1L, or metabolized to THF and released as a CO_2_ via ALDH1L2. Formate is present in mitochondria and is used as substrate for the bidirectional enzyme MTHFD1 to form 10-formyl-THF, for de novo purine synthesis, and 5,10-methylene-THF for thymidylate synthesis as well as for the methionine cycle. Several inhibitors have been identified that target one carbon metabolism, including methotrexate and aminopterin for DHFR, SHIN1/2 for SHMT1/2, and LY345899 and DS18561882 for MTHFD1/2. Abbreviations: DHF dihydrofolate, THF tetrahydrofolate, DHFR dihydrofolate reductase, SHMT1/2 serine hydroxymethyltransferase 1/2, THF tetrahydrofolate, MTHFD2 methylenetetrahydrofolate dehydrogenase 2, MTHFD1/2L methylenetetrahydrofolate dehydrogenase 1/2 like, ALDH1L2 aldehyde dehydrogenase 1 family member L2 also known as 10-formyl-THF dehydrogenase, TYMS thymidylate synthase.

One carbon metabolism is a group of connected cytosolic and mitochondrial reactions that comprise the methionine and the folate cycles (Fig. 3). These reactions provide one carbon units in the form of methyl groups to several metabolic pathways and are responsible for the synthesis of thymidine, methionine, serine/glycine, and purine [55]. While the methionine cycle occurs in the cytosol, folate-dependent one carbon metabolism synthesizes serine/consumes glycine in the cytosol and catabolizes serine/produces glycine in mitochondria [56].

Aminopterin, a folate analog, was one of the first chemotherapeutic agents inducing leukemia remission [57]. It is a precursor of methotrexate, which is still widely used today for treatment of cancer as well as other diseases. Methotrexate is an inhibitor of dihydrofolate reductase, synthesizing tetrahydrofolate, the active form of folic acid. The products derived from methotrexate intracellular metabolism, polyglutamates, also inhibit two of the enzymes involved in purine synthesis [58].

Upregulation of the mitochondrial folate and serine/glycine enzymes is associated with increased sensitivity to methotrexate in different tumor types [59]. High levels of the enzymes participating in the mitochondrial branch of the folate cycle, serine hydroxymethyltransferase 2 (SHMT2), methylenetetrahydrofolate dehydrogenase 2 (MTHFD2), and monofunctional tetrahydrofolate synthase 1L (MTHFD1L), have been linked to rapid proliferation in the NCI-60 panel of cancer cell lines [60]. *Serine hydroxymethyltransferase 2 (SHMT2)* and *MTHFD2* were indeed among the most overexpressed metabolic genes in nineteen different cancer types analyzed [61]. Increased levels of MTHFD2 are related to breast cancer cell migration and invasion [40] and show correlation with markers of poor prognosis [62]. Upregulation of *SHMT2*, *MTHFD2*, and *ALDH1L2* (10-formyltetrahydrofolate dehydrogenase) were found to be associated with poor survival in colorectal cancer [63]. The enzymes of the mitochondrial serine/glycine metabolism are elevated in primary NSCLC tumor initiating cells, specifically, glycine decarboxylase (GLDC). High levels of GLDC are also identified in other primary tumors, especially ovarian and germ cell tumors [64].

These data support the role of mitochondria as important regulators of cell proliferation through the folate-serine/glycine pathway, and have led to the development of novel inhibitors targeting mitochondrial one carbon metabolism [65]. However, the high sequence homology of the mitochondrial enzymes MTHFD2 and SHMT2 with their cytosolic counterparts, MTHFD1 and SHMT1, complicates precision medicine approaches. Only two compounds inhibiting MTHFD2, LY345899 [66] and DS18561882 [67], have shown anticancer activity both in vitro and in vivo. Knockdown experiments of *SHMT2* in colorectal cancer xenografts show total blockage of tumor development only when SHMT1 is also downregulated [68]. Many of the inhibitors developed target both SHMT1 and SHMT2 due to their high sequence identity. They include derivatives from herbicidal compounds called pyrazolopyrans, used as antimalarial drugs [69]. Optimization has generated several dual SHMT1/2 inhibitors, including SHIN1 [68] and SHIN2 [70]. While SHIN1 has poor stability and short half-life in vivo, SHIN2 potently inhibits T cell acute lymphoblastic leukemia xenograft growth, and shows synergistic effects with methotrexate [70]. In addition, several folate analogs inhibit SHMT, such as LTX, which may be further optimized [71, 72].

### Fatty Acid β-Oxidation (FAO)

**Fig. 4:**
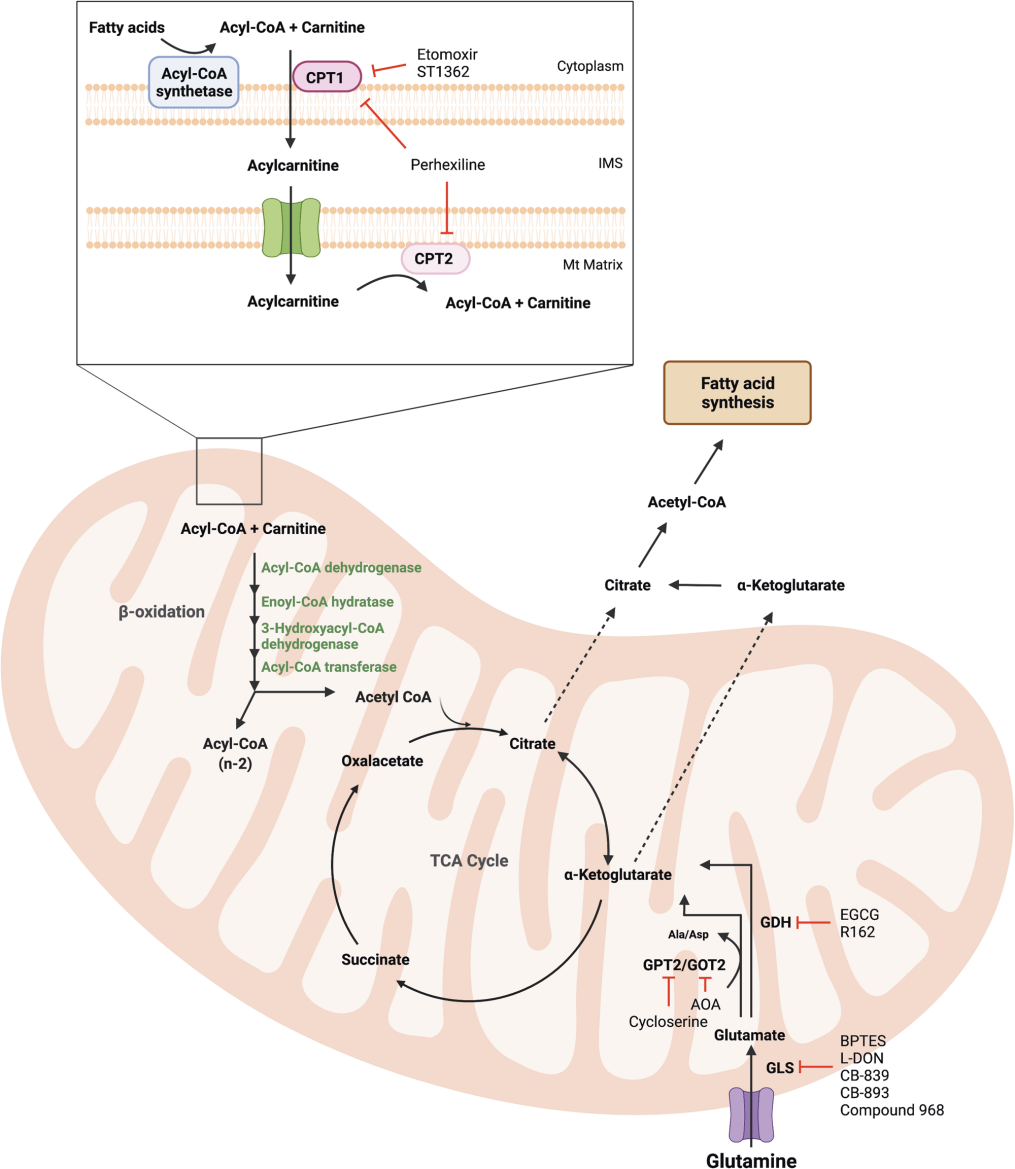
Inhibitors of glutamine metabolism and β-oxidation (FAO). Glutaminase (GLS) converts glutamine to glutamate, which is further metabolized to α-KG by GDH. In turn, α-KG can be converted to citrate. Both α-KG and citrate can be exported to the cytosol for use as precursors for de novo fatty acid synthesis. Glutamate can also be metabolized by GPT2/GOT2 aminotransferases, producing alanine/aspartate and α-KG. Several inhibitors of glutamine metabolism have been described, including BPTES, CB-830, CB-893, and compounds 968 for GLS, EGCG and R162 for GDH and AOA, and cycloserine for GOT2 and GPT2, respectively. For β-oxidation (FAO), fatty acids need to be transferred to mitochondria by the action of CPT1/2. The most used inhibitors of FAO are etomoxir and teglicar (ST1326), both targeting CPT1. Moreover, perhexiline acts as a dual CPT1/2 inhibitor. Abbreviations: α-KG, α-ketoglutarate; GLS, glutaminase; GDH, glutamate dehydrogenase; GPT2, mitochondrial glutamate-pyruvate transaminase 2; GOT2, aspartate aminotransferase; EGCG, epigallocatechin-3-gallate; AOA, amino-oxyacetic acid; IMS, intermembrane mitochondrial space.

Fatty acids are energy rich nutrients, providing more than twice as much ATP as carbohydrates or proteins per gram of dry mass [73]. Fatty acid oxidation (FAO), also called β-oxidation, is a cyclic series of catabolic reactions performed by a trifunctional enzyme complex located in the mitochondrial matrix (Fig. 4 and Supplementary Box 8).

This pathway is a source for high levels of ATP and it is activated to sustain high proliferation and to cope with stress in many tumors [74]. Normally, cells displaying fatty acid synthesis (FAS) suppress FAO through inhibition of carnitine palmitoyltransferases (CPT1) by malonyl-CoA produced by acetyl carboxylase 1 or 2 (ACC1 and ACC2). While ACC1 is a cytoplasmic enzyme of de novo fatty acids synthesis, ACC2 is located in mitochondria, and is involved in inhibition of FAO by producing malonyl-CoA in the proximity of CPT1 [75]. However, in some cancers, elevated FAO also provides high ATP levels. This occurs in cells that simultaneously engage in FAS, through epigenetic mechanisms that allow a differential expression of *ACC1* and *ACC2*, resulting in selective *ACC2* downregulation [74].

The most common inhibitors of FAO target the rate-limiting CPT1 enzyme. *CPT1A* (the liver isoform) is upregulated in high-grade serous ovarian cancer (HGSOC) and in acute lymphoblastic leukemia, and correlates to poor overall survival [76]. Etomoxir, the most utilized CPT1 inhibitor, prevents tumor progression in a model of HGSOC [77]. Elevated levels of *CPT1A* are associated with poor prognosis in acute lymphoblastic leukemia [76]. SRC and MYC-driven FAO supports triple-negative breast cancer (TNBC) [78, 79]. Etomoxir blocks tumor growth and metastatic features of breast cancer patient-derived xenografts (PDXs) [78], in MYC-driven transgenic TNBC mouse models, and in *MYC*-overexpressing PDXs [79]. Notably, we found that FAO is the main energy source in *MYCN*-amplified neuroblastoma and that etomoxir reduces in vitro proliferation and tumor burden in vivo [80]. Etomoxir further induces cell death of glioblastoma cells in vitro [81] and delays tumor apparition and progression in a glioblastoma mouse model [82]. However, toxicity, off-target effects on ETC Complex I, and the high doses needed, limits its potential for clinical applications. This has led to the development of the more selective CPT1 inhibitor teglicar (ST1326) [83] and the dual CPT1-CPT2 inhibitor perhexilline [84]. 3-Ketoacyl-CoA thiolase, the final FAO enzyme, is inhibited by trimetazidine and ranolazine [85, 86], both approved for the treatment of angina pectoris [87]. However, no FAO inhibitory activity was observed either in cell lines, in primary cells, or in mice [88]. Thus, more specific, and less toxic FAO inhibitors are needed to exploit lipid oxidation as a precision medicine target in cancer.

### Glutamine metabolism

Glutamine is the most abundant amino acid in human plasma and is the main form of nitrogen transportation between organs. Although considered a non-essential amino acid, it is required for the synthesis of other amino acids, proteins, nucleotides, glutathione, and is involved in the activation of mTORC1 [17]. Numerous cancer cells are considered “glutamine addicted” and use glutamine as an anaplerotic source for the TCA cycle (Supplementary Box 9).

The first glutamine analogs reported were 6-diazo-5-oxo-L-norleucine (L-DON), azaserine [89, 90], and acivicin [91] but they had limited clinical applications due to toxicity [92]. Genetic silencing or the inhibition of glutaminase (GLS) using the allosteric inhibitor BPTES [93] decreases proliferation, induces cell death, and reduces proliferation in cancer cell lines as well as tumor growth in vivo [94, 95]. CB-839 is another GLS inhibitor that underwent clinical trials for treatment of several hematological tumors, colorectal cancer, melanoma, ccRCC, NSCLC, TNBC, and others (Fig. 4) [96].

High glutamate dehydrogenase (GDH) leads to increased α-KG synthesis, inducing glutamine-derived carbon flux into the TCA cycle and promoting anaplerosis and energy production. In addition, elevated GDH levels contribute to increased fumarate,that in turn, binds and activates the glutathione peroxidase enzyme, enhancing ROS detoxification in myeloma, leukemia, breast, and lung cancer cell lines. Glioblastoma cells show a strong dependency on GDH [97] and it is also essential for ammonia recycling in breast cancer cells, as it supports the high demand for amino acid synthesis [98]. Two GDH inhibitors are epigallocatechin-3-gallate, with low specificity [99], and R162, which impairs ROS detoxification and reduces glioma growth in vitro and in vivo [100]. Additional inhibitors of GDH are hexachlorophene and bithionol [101], but their anticancer potential is unexplored.

Aspartate aminotransferase 2 (GOT2) promotes ATP production and ROS homeostasis, supporting pancreatic tumor growth in vivo [102]. Pancreatic ductal adenocarcinoma depends on both cytosolic (GOT1) and mitochondrial aspartate aminotransferases [103]. The *GOT2* gene is repressed by the tumor suppressor BRCA1 in breast cancer cells, and related to poor survival in TNBC patients [104]. Despite the relevance of transaminases in cancer, only few inhibitors are available. Amino-oxyacetic acid, an inhibitor of GOT1/GOT2 and other transaminases, diminishes oxygen consumption, reduces the growth of breast cancer cells and xenografts [105], and induces senescence in pancreatic cancer cells [106]. However, this compound is not specific enough for use as an anticancer drug.

Mitochondrial glutamate-pyruvate transaminase 2 (GPT2) is upregulated upon glutamine deprivation or GLS inhibition with BPTES or CB-839 in a process that involves ROS production and ATF4 (activating transcription factor 4) [107]. The combination of BPTES with the GPT2 inhibitor cycloserine [108] shows synthetic lethality in multiple cancer cell lines [107]. In breast cancer, its high expression is correlated to the pathological grade, as it promotes tumorigenesis and stemness [109]. Given the roles of GDH, GOT2, and GPT2 in cancer cells, further efforts should be invested to develop specific and potent inhibitors for these enzymes.

## Mitochondrial ROS

**Fig. 5:**
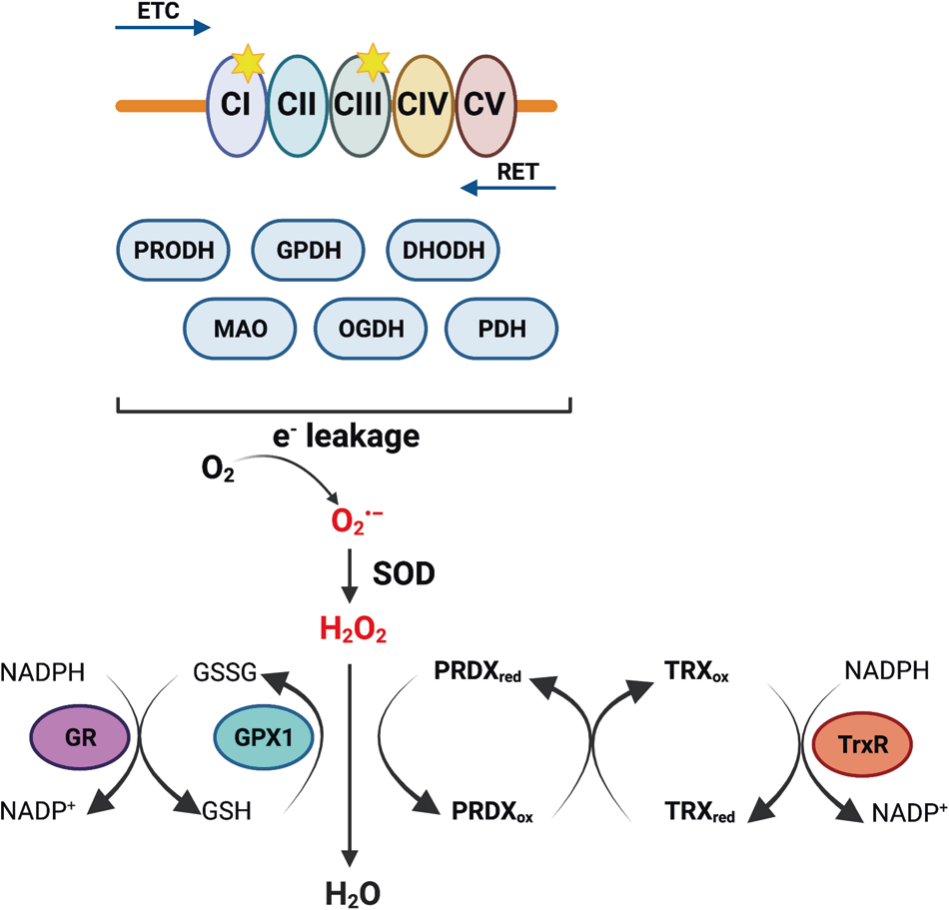
Mitochondrial ROS and antioxidant systems. Electrons can leak from the ETC, especially from Complex I and III (marked with yellow stars), from the reverse electron transport chain (RET) or mitochondrial enzymes as proline dehydrogenase (PRODH), glycerophosphate dehydrogenase (GPDH/GPD2), mitochondrial dehydroorotate dehydrogenase (DHODH), mono-amino oxidase (MAO), 2-oxoglutarate dehydrogenase (OGDH), and pyruvate dehydrogenase (PDH). This electron leakage can result in the formation of superoxide radical (O_2_^•–^), which superoxide dismutases (SOD) convert it into hydrogen peroxide (H_2_O_2_). H_2_O_2_ will be processed into water (H_2_O) by the antioxidant systems. The glutathione system includes the glutathione (GSH) peroxidase (GPX1) and the glutathione disulfide (GSSG) reductase (GR). The peroxiredoxin-thioredoxin system alternates between reduced to oxidized peroxiredoxin (PRDX) and thioredoxin (TRX), and thioredoxin reductase (TrxR), which catalyzes the NADPH-dependent reduction of TRX.

Reactive oxygen species (ROS) are intracellular oxygen species (mainly, O_2_^•–^, H_2_O_2,_ and OH˙). Their levels are tightly regulated in the cell, as an appropriate balance sustains signal transduction while avoiding cellular damage. Mitochondria are a major source of ROS, as they can be produced along the ETC but also by the reverse electron transport (RET; Supplementary Box 10) and different enzymatic reactions (Fig. 5 and Supplementary Box 11) [110].

Increased ROS levels are a common feature in cancer and can be pro-tumorigenic by enhancing survival, proliferation, migration, invasion, and genetic instability, but also anti-tumorigenic when the levels cross the threshold that leads to high oxidative stress and cell death. Therefore, tumor cells express oncogenes that potentiate ROS production, and, at the same time, increase their antioxidant systems or decrease OXPHOS. Thus, achieving optimal ROS levels is a driver of cancer, known as the “ROS rheostat theory” [111, 112].

Oncoproteins can mediate an increase in ROS by two mechanisms: (i) increased TCA cycle fueling and mitochondrial mass and (ii) destabilization of the ETC utilized by KRAS, MYC, PI3K-AKT-mTOR, and BCR/ABL [110]. KRAS and MYC induce nuclear factor erythroid 2-related factor 2 (NRF2), activating the expression of genes from the antioxidant pathways, such as SODs, glutathione peroxidase, glutathione reductase, peroxiredoxin, thioredoxin, and catalase. Tumor cells can also use glutamine for its antioxidant capacity, as it is the precursor of glutathione, generating NADPH through the malate pathway, necessary for detoxification reactions. Another process is the modification of cancer cell metabolism to optimize ROS. Highly proliferative cancer cells with glucose abundance are glycolytic to avoid ROS production while still producing energy. In OXPHOS-dependent tumors, elevated respiration and high ROS are compensated by expression of antioxidant enzymes [112, 113]. The increase in ROS also leads to activation of other signaling pathways related to tumorigenesis, such as PI3K, MAPK, and HIFs [114].

Several drugs, including inhibitors of the ETC, induce ROS through different mechanisms. Therefore, evaluating the ROS baseline levels of a tumor could be useful to determine the responsiveness to ROS-inducing agents. It could be combined with inhibitors of the compensatory mechanisms, i.e., glycolysis or the antioxidant proteins [113]. Inhibitors of the glutamine pathway impair the formation of glutathione, and thus dysregulate the antioxidant system. In addition, glycolysis can be suppressed by 2-deoxyglucose or dichloroacetate. The glutathione system can be inhibited with NOV-002, L-buthionine-S, R-sulfoximine, canfosfamide, or ezatiostat hydrochloride. Sulfasalazine, an inhibitor of the cysteine/glutamate antiporter XCT, showed efficacy in pancreatic and SCLC cells [113]. The thioredoxin system is inhibited by PX-12, SAHA, PMX464, auranofin, and motexafin gadolinium [115], the 2-Cys peroxiredoxins by conoidin A [116], and peroxiredoxin 6 by MJ33 [117]. NOV-002 is a glutathione disulfide mimetic that induces oxidative stress and has been used in clinical trials for breast, ovarian, and NSCLC [118]. PX-12 has enrolled clinical trials for refractory cancers [119] while ATN-224, a SOD1 inhibitor, is in clinical trials for prostate cancer [120].

## Ketogenic diet and mitochondria

Ketogenic diet is high in fat and low in carbohydrates, normally in a 4:1 fat/non-fat ratio [121]. The use of fat as a main source of energy mimics the metabolic state of starvation and the body switches to FAO as the main energy source. Fat is catabolized to ketone bodies in mitochondria of hepatocytes, consisting of 3-β-hydroxybutyrate (βOHB), acetoacetate, and acetone (Supplementary Box 12) [122].

This diet was first used as an anticancer approach in two patients with malignant brain cancer that did not respond to radiation and chemotherapy. Ketone blood levels increased 20–30-fold and the patients showed improved outcomes [123–125]. Some studies have reported that cancer cells have lower levels of succinyl-CoA-ketoacid-CoA transferase (SCOT) [126], making them unable to use βOHB as energy fuel. The increase in ketones can impair cancer cell growth while maintaining the functions of healthy tissues [122, 127]. MYC represses 3-hydroxymethylglutaryl-CoA synthase (*HMGCS2*) in colon cancer [128] and decreased expression of *HMGCS2* is found in ccRCC [129] and hepatocellular carcinoma [130], associated with worse patient outcome. Inactivation of hydroxymethylglutaryl-CoA lyase resulted in enhanced proliferation and metastasis of nasopharyngeal carcinoma [131].

Importantly, not all studies applying ketogenic diet to cancer show a clear effect, suggesting that the type of cancer, genomic profile, as well as time and characteristics of the diet result in different outcomes. A combination of drug treatment and ketogenic diet may be an attractive approach to potentiate therapeutic response, as demonstrated in multiple clinical trials [132]. An alternative is calorie restriction, which apart from lowering glucose in blood and insulin, also decreases lipid levels. In models of pancreatic ductal adenocarcinoma and NSCLC, calorie restriction but not ketogenic diet resulted in reduced tumor progression [133].

## Mitochondrial metabolism and immunotherapy

Mitochondria have crucial roles in CD8^+^ T cell differentiation, maintenance, and their functional decline in the TME. The efficacy of immunotherapy could be increased in combination with mitochondria-modulating treatments. PD-1 blockade therapy-responsive tumors have higher basal respiration, maximal respiration, spare respiratory capacity, and ATP turnover. The reduction in mitochondrial activity in T cells may explain the escape from PD-1 blockade therapy in some tumors [134]. Bezafibrate, an agonist for peroxisome proliferator-activated receptor gamma coactivator 1-alpha (PGC1α), has been shown to boost antitumor immunity by upregulating mitochondrial OXPHOS and inhibiting apoptosis in an MC38-bearing mouse tumor model treated with PD-1 blockade [135]. The 4-1BB costimulatory receptor expressed on activated T and natural killer cells, augments glycolysis upon induction, by promoting the expression of glucose transporters to support CD8^+^ T cell proliferation [136]. Moreover, in melanoma tumors with high oxidative metabolism, and low glycolysis, PD-1 blockage correlated with poor response in patients [137]. All these studies support the importance of combination strategies with mitochondrial metabolic inhibitors to combat resistance to immunotherapy.

## Future perspectives

One hundred years ago, Otto Warburg proposed that altered metabolism is a characteristic of cancer cells. A decade ago, metabolic reprogramming was recognized as one of the hallmarks of cancer, as tumor cells need to maintain a high rate of macromolecular synthesis, essential for cell growth and division. During the last years, advances have highlighted the importance of OXPHOS, FAO, as well as glutamine addiction in cancer cells, setting the focus on mitochondrial metabolism. Many tumor types harbor mutations in rate-limiting enzymes regulating major mitochondrial metabolic pathways. Some of these alterations result in accumulation of oncometabolites acting as epigenetic regulators, or in the increase of ROS production, contributing to tumorigenesis. Importantly, the significant role of mitochondrial metabolism in tumorigenesis could potentially be exploited as a strategy for cancer therapy. The successful use of the anti-folates aminopterin and methotrexane in clinical practice, with remission of acute lymphocytic leukemia in children, provided evidence that targeting metabolism represents an effective therapeutical approach. Notably, several compounds inhibiting mitochondrial metabolic enzymes have been identified, some of which, like etomoxir and BPTES, showed promising effects in vitro. However, translation from preclinical experiments has been challenging, with only few compounds currently in early-stage clinical trials (Table 1). Next-generation technologies, including metabolic profiling and single-cell sequencing of human tumors, as well as metabolic tracing of tumors in mice will provide novel knowledge of the mechanisms regulating mitochondrial metabolism in different cancer types. Identification of the most appropriate strategy for each patient will result in more specific and less toxic treatments. Importantly, the combination of standard chemotherapeutic drugs, immunotherapy, or ketogenic diet with mitochondrial inhibitors may be an attractive, efficient approach for cancer cure. The future looks bright and holds promise for discovery of novel remedies for precision medicine.

**Table 1:** Mitochondrial enzymes for clinical application.

Pathway	Enzyme	Inhibitors	Limitations	References
TCA cycle	IDH (mutant)	AGI-5198, AG-221, AG-881	Few inhibitors described	[33–35]
	KGDHC	CPI-613	Few inhibitors described	[36, 37]
	PDC	CPI-613, PDT-PAO-16	Few inhibitors described	[36, 37, 138]
ETC	Complex I	Tamoxifen, metformin, IACS-010759	Need to evaluate anti-tumor properties in more tumor types	[49, 139, 140]
		Rotenone, MPP^+^, deguelin NRTIs	Experimental compounds Used for HIV treatment; need for testing anti-cancer properties	
	Complex II	Malonate, nitropropionic acid, TTFA, troglitazole, 3-bromopyruvate, α-tocopherol succinate	Experimental compounds	[49]
	Complex III	Resveratrol	Lack of specificity	[141]
	Complex IV	Doxorubicin, Photofrin, Fenretinide	Few inhibitors described against the human protein	[52, 142, 143]
	Complex V	ND	Lack of inhibitors with clinical application	[49]
One carbon metabolism	SHMT2	Pyrazolopyrans: SHIN1; SHIN2 Folate analogs: LTX; methotrexate; pemetrexed; raltitrexed 4-chloro-L-threonine beta-trifluoroallothreonine substituted hydroxylamine derivates sulfonyl fluoride triazine derivates	Lack of specificity	[68–72]
	MTHFD2	LY345899; DS18561882	Lack of specificity Few inhibitors described	[66, 67]
	ALDH1L2	ND	No inhibitors described against the human protein	
	GLDC	ND	No inhibitors described against the human protein	
Fatty acid oxidation	CPT1	Etomoxir ST1326 Perhexiline C75-CoA CoA esters of oxirane carboxylic acids Octyl glucoside	Toxicity Lack of specificity	[144–148]
	acyl-CoA dehydrogenase	ND	No inhibitors described against the human protein	
	enoyl-CoA hydratase	ND	No inhibitors described against the human protein	
	3-hydroxyacyl-CoA dehydrogenase	ND	No inhibitors described against the human protein	
	acyl-CoA acetyltransferase	Trimetazidine Ranolazine	Inhibitory activity could not be confirmed in replication studies	[85, 88]
Glutamine-related processes	GLS (2 isoforms: KGA and GAC)	BPTES (KGA) DON (all GLS and GLS2 isoforms) Azaserine (all GLS and GLS2 isoforms) CB-839 (KGA and GAC) N-(5-{2-[2-(5-amino-[1,3,4]thiadiazol-2-yl)-ethylsulfanyl]-ethyl}-[1,3,4]thiadiazol-2-yl)-2-phenyl-acetamide 6 (BPTES analog) bis-2-(5-phenylacetimido-1,2,4,thiadiazol-2-yl)ethyl sulfide (GAC) Compound 968 (GAC)	Toxicity Lack of specificity Low affinity and potency Low bioavailability and stability in plasma	[89, 90, 93, 149–152]
	GLS2 (Two isoforms, GAB and LGA)	DON (all GLS and GLS2 isoforms) Azaserine (all GLS and GLS2 isoforms) Alkyl benzoquinones (GAB)	Toxicity Lack of specificity Low affinity and potency No inhibitors described for LGA	[89, 153]
	GDH	R162 Hexachlorophene Bithionol EGCG	Few inhibitors described for the human protein Inhibitors not tested for anti-cancer properties Lack of specificity	[99–101]
	GOT2	Amino-oxyacetic acid	Lack of specificity Few inhibitors described	[105, 106]
	GPT2	Amino-oxyacetic acid Cycloserine Vigabatrin	Need for testing anti-cancer properties Lack of specificity Few inhibitors described	[107, 108, 154]

Enzymes are classified in metabolic pathways with available inhibitors specified, their main limitations, and references from Pubmed and Brenda database (brenda-enzymes.org).

Abbreviations: TCA, tricarboxylic acid; IDH, isocitrate dehydrogenase; KGDHC, α-ketoglutarate dehydrogenase complex; PDC, pyruvate dehydrogenase complex; MPP^+^, methyl-4-phenylpyridinium; NRTIs, Nucleoside analog reverse transcriptase inhibitors; TTFA, thenoyltrifluoro-acetone; ETC, electron transport chain; SHMT2, serine hydroxymethyltransferase 2; SHIN1/2 SHMT, inhibitor 1/2; LTX, lometrexol; MTHFD2, methylenetetrahydrofolate dehydrogenase 2; ALDH1L2, Aldehyde Dehydrogenase 1 Family Member L2; GLDC, glycine decarboxylase; CPT1, carnitine palmitoyltransferase 1; GLS, glutaminase; KGA, kidney-type glutaminase; GAC, glutaminase C; GDH, glutamate dehydrogenase; EGCG, Epigallocatechin gallate; GOT2, aspartate aminotransferase; GPT2, mitochondrial glutamate-pyruvate transaminase 2; ND, not determined.

## Supplementary information

Supplemental Material

## Data Availability

This review article does not present any new primary data.
