# Supplementary material for: Targeting mitochondrial metabolism for precision medicine in cancer
Source: Cell Death Differ. 2022 Jul 13;29(7):1304–17. doi: 10.1038/s41418-022-01022-y (PMC9287557; doi:10.1038/s41418-022-01022-y)
Supplement: Supplementary file 1 — Supplemental Material [file 41418_2022_1022_MOESM1_ESM.docx]

Supplementary Material

Targeting Mitochondrial Metabolism for Precision Medicine in Cancer

Running title: Mitochondrial metabolism in precision cancer therapy

Lourdes Sainero-Alcolado^1,2^, Judit Liaño-Pons^1,2^, María Victoria Ruiz-Pérez^1^, and Marie Arsenian-Henriksson^1*^

^1^ Department of Microbiology, Tumor and Cell Biology (MTC), Biomedicum B7, Karolinska Institutet, SE-171 65 Stockholm, Sweden. L.S-A, lourdes.sainero.alcolado@ki.se; J.L-P, judit.liano.pons@ki.se; M.V.R-P, maria.ruiz.perez@ki.se; M.A-H, marie.arsenian.henriksson@ki.se

^2^ These authors contributed equally to this work

***** Correspondence: marie.arsenian.henriksson@ki.se; Tel.: +46852486205

**Supplementary Table 1. Main Metabolic Pathway of Tumors.** Tumor type and main metabolic pathways for ATP production, Glycolysis or OXPHOS, are specified together with the main fuel used, as well as related mutations if described. Abbreviations: OXPHOS, oxidative phosphorylation; AML, acute myeloid leukemia; CSC, cancer stem cells; *MYCN*-amp, *MYCN*-amplified; ETC, electron transport chain; CI, complex I; DLBCL, diffuse large B-cell lymphoma; SCLC, small cell lung cancer; MGPDH, Mitochondrial glycerophosphate dehydrogenase; PGC-1α, peroxisome proliferator-activated receptor (PPAR) γ coactivator 1-α.; ccRCC, clear cell renal cell carcinoma; FH, fumarate hydratase; HLRCC, hereditary leiomyomatosis and renal cell cancer; VHL, von Hippel-Lindau; SDH, succinate dehydrogenase; ND, not determined.

| Tumor type | Metabolic profile | Main fuel | Related mutations |
| --- | --- | --- | --- |
| Melanoma | OXPHOS | ND | Increase of PGC1α |
| Melanoma: BRAF activating mutation | OXPHOS | ND | Activating mutation in BRAF and increase of PGC1α |
| Leukemic cells driven by BCR-ABL | OXPHOS | ND | BCR-ABL |
| Leukemic stem cells from AML | OXPHOS | Amino acids and fatty acids | ND |
| Peritoneal cancer  (serous adenocarcinoma) | OXPHOS | ND | ND |
| Ovarian cancer  (serous adenocarcinoma) | OXPHOS | ND | ND |
| Glioma cell lines  (D-54MG and GL261) | OXPHOS | Glucose | ND |
| Glioma cell lines  (U-251MG and U-87MG) | Glycolytic | Glucose | ND |
| Glioblastoma | Glycolytic (main) + OXPHOS | Glucose | ND |
| Epithelial ovarian cancer: CSC (patients) | OXPHOS | Glucose | ND |
| *MYCN*-amp Neuroblastoma | Glycolytic + OXPHOS | Fatty acids | *MYCN*-amp |
| Prostate cancer | OXPHOS | Switch from glutamate/malate to succinate driven OXPHOS in high grade tumors | ETC CI mutations |
| DLBCL | OXPHOS | Palmitate | Non-functional BCR signaling |
| SCLC | Glycolytic | Glucose | *MYC* overexpression |
| Thyroid cancer | OXPHOS | Glucose | *MGPDH* overexpression |
| Cholangiocarcinoma  stem cells | OXPHOS | ND | *PGC1α* overexpression |
| Cholangiocarcinoma tumor cells | Glycolytic | ND | ND |
| Pancreatic ductal adenocarcinoma | Glycolytic | Glucose | *KRAS* |
| ccRCC | Glycolytic | Glucose | *VHL* loss of function |
| HLRCC | Glycolytic | Glucose | *FH* loss of function |
| Hereditary paraganglioma | Glycolytic | Glucose | *SDH* loss of function |

**Supplementary Table 2. Genes encoding for TCA cycle enzymes altered in cancer.** Gene, type of alteration (mutation or differential expression), together with the consequences for the cell, as well as tumor types are specified. Abbreviations: D-2HG, D-2-hydroxyglutarate; AML, acute myeloid leukaemia; B-ALL, B-cell acute lymphoblastic leukaemia; T-ALL, T-cell acute lymphoblastic leukaemia.

| Gene | Type of alteration | Consequences | Tumour type |
| --- | --- | --- | --- |
| *ACO2* | Downregulation | Export of large amounts of citrate | Gastric cancer |
| *CS* | Upregulation | Increase in *de novo* fatty acid synthesis | Pancreatic, ovarian, and prostate cancer |
| *IDH1/2* | Gain of function mutation | Production of oncometabolite  D-2HG from α-KG  ﻿Increased affinity for NADPH/α-KG but reduced for isocitrate | Glioma, glioblastoma, AML, B-ALL, angioimmunoblastic T-cell lymphoma, chondrosarcoma, intrahepatic cholangiocarcinoma, myelodysplastic syndromes, thyroid carcinoma, prostate carcinoma |
| *FH* | Loss of function mutation or downregulation | Abnormal accumulation of fumarate  Stabilization of HIF1α  Glutamine used for heme synthesis and and as a main fuel | Pheochromocytoma, paraganglioma, neuroblastoma,  renal cell cancer, type 2 papillary renal cell carcinoma, multiple and hereditary leiomyomatosis, ﻿Leydig cell tumor, ﻿ovarian mucinous cystadenoma |
| *SDH* | Loss of function mutation or downregulation | Abnormal accumulation of succinate.  Stabilization of HIF1α  Impairment of complex II | Pheochromocytoma, paraganglioma, colorectal cancer, renal cell carcinoma, gastrointestinal stromal tumor, pituitary carcinoma, T-ALL |
| *SDHAF2* | Loss of function mutation | Loss of SDH function and stability | Hereditary head and neck, paraganglioma |
| *KGDHC* | Upregulation | Increase of mitochondrial bioenergetics  Increase expression of EMT transcription factors | Gastric cancer |

Supplementary Figure 1


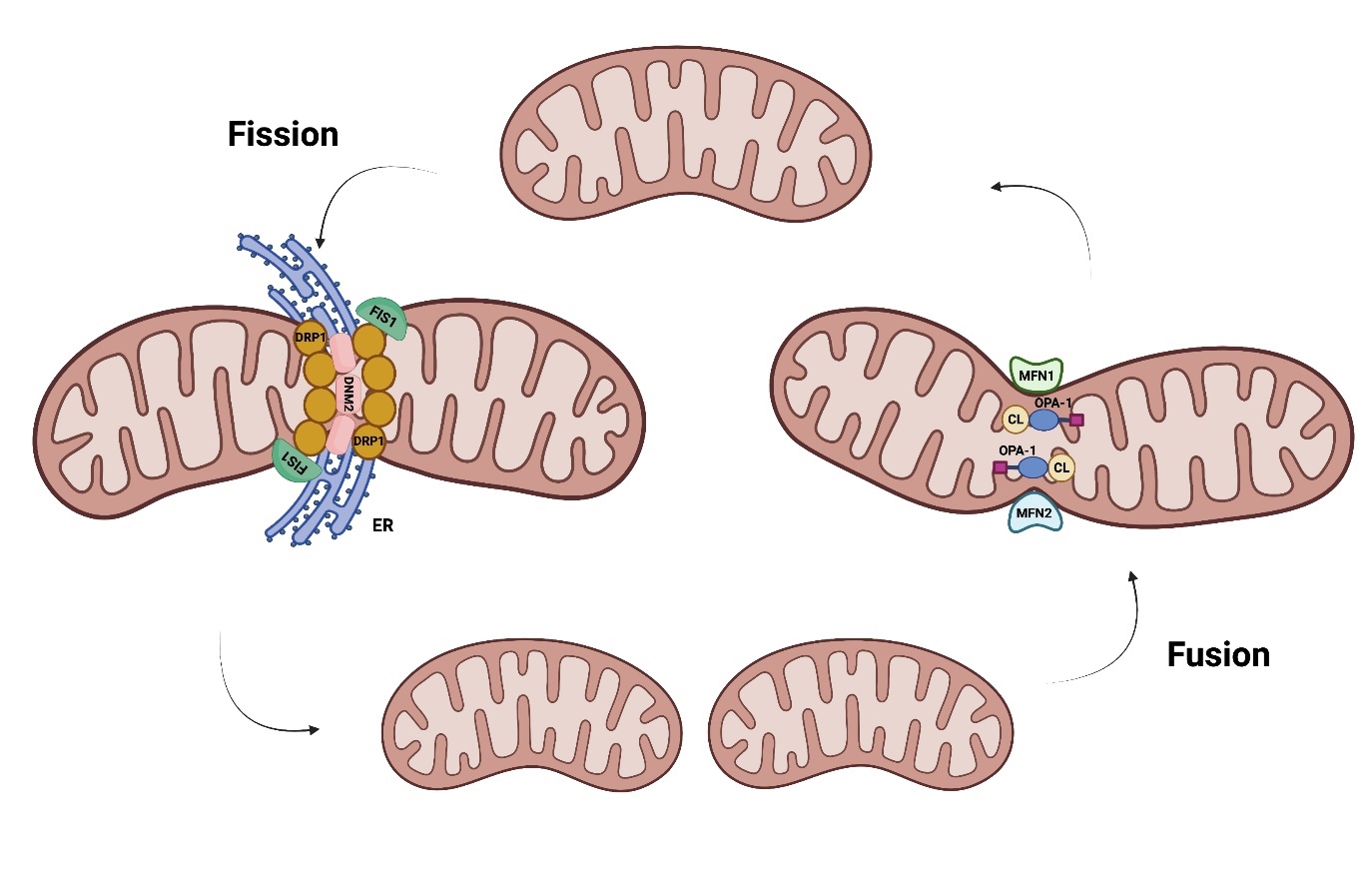


**Supplementary Figure 1. Mitochondrial dynamics.** Fission and fusion are key processes in mitochondrial dynamics. Fission starts with replication of mtDNA in the matrix, stabilizing the site for endoplasmic reticulum (ER) recruitment. Dynamin-related/-like protein 1 (DRP1) is recruited by fission 1 (FIS1) and the constriction starts at the mitochondria-ER contact sites. DRP1 oligomerizes and forms a ring structure around the place of division. Next, dynamin2 (DNM2) finishes the fission into two mitochondria. Fusion is driven by the GTPases mitofusin 1 (MFN1) and mitofusin 2 (MFN2) and the optic atrophy 1 (OPA1) enzyme. The process starts by the outer mitochondrial membrane (OMM) tethering of both mitochondria, followed by the conformation change of MFN1 and MFN2, which facilitates OMM fusion. Inner mitochondrial membrane (IMM) fusion is carried out by OPA1 and cardiolipin interaction and OPA1-dependent GTP hydrolysis.

**Supplementary Box 1**

**Mutations Involved in the Metabolic Reprogramming of Cancer Cells**

Amplification or overexpression of *MYC* enhances the levels of transporters and enzymes involved in glycolysis, genes responsible for glutamine uptake and glutaminolysis, fatty acid synthesis, mitochondrial biogenesis, and serine metabolism. Tumors that reside in a low oxygen environment (hypoxia) suffer a metabolic adaptation regulated by HIF-1, which in turn increases the glycolytic function. Constitutive activation of HIF-1α in normoxia has been observed in several tumors as a result of mTOR activation, loss of the *von Hippel-Lindau* (*VHL*) gene (responsible for HIF-1α proteasomal degradation in normoxia), and mutations in the genes encoding for tricarboxylic acid (TCA) cycle enzymes succinate dehydrogenase (*SDH*) and fumarate hydratase (*FH*) or reactive oxygen species (ROS) accumulation. Similarly to its downstream effector MYC, RAS promotes an increase in enzymes participating in glycolysis and glutaminolysis. The p53 tumor suppressor is altered in approximately 50% of all cancers. Apart from its role in DNA repair, cell cycle arrest, and apoptosis, p53 also participates in metabolic control by inhibiting glycolysis and increasing OXPHOS. PTEN negatively regulates the PI3K-AKT-mTOR pathway by dephosphorylating phosphatidyl-inositol 3,4,5-trisphosphate (PIP3), the second messenger produced by PI3K. Moreover, PTEN decreases glucose and glutamine uptake and increases mitochondrial ATP production.

**Supplementary Box 2**

**Mitochondrial DNA**

Years of evolution have resulted in a mitochondrial genome composed by 16.5 kb encoding 37 genes crucial for OXPHOS activity: 13 encoding essential components of the OXPHOS machinery, 22 transfer RNAs necessary for mitochondrial translation, and the 12S and 16S ribosomal RNAs. Therefore, a high number of mutations in mtDNA results in impaired energy production. However, most of the approximately 1,000 mitochondrial proteins are encoded by nuclear DNA (nDNA), synthesized, and imported from the cytoplasm into the organelle. The mutagenesis rate of mtDNA is 10-20-fold higher than nDNA due to its lack of introns and histones, the proximity to a more mutagenic environment in which ROS are forming, a less efficient DNA repair mechanism, and its replication in an asymmetrical manner, with parts of single-stranded DNA prone to spontaneous nucleotide deamination.

**Supplementary Box 3**

**Mitochondrial Dynamics**

- 1. Mitogenesis

Regulation of mitochondrial mass homeostasis is crucial for healthy cells, and requires the coordination of two opposite processes, mitogenesis and mitophagy, respectively. In situations of increased energy demand and other environmental stress factors, mitochondrial biogenesis (*i.e*. mitogenesis) is activated. New mitochondria are formed from already existing ones, in a self-renewal manner. The key regulator of mitogenesis is peroxisome proliferator-activated receptor (PPAR) γ coactivator 1-α (PGC-1α), which interacts with the nuclear respiratory factors 1 and 2 (NRF-1 and NRF-2), and the mitochondrial transcription factor A (TFAM). NRF-1, NRF-2, and TFAM regulate expression of genes encoding key mitochondrial enzymes involved in the respiratory complex, mitochondrial protein import machinery, and mtDNA synthesis, among others. Moreover, the cyclic adenine monophosphate and protein kinase A (cAMP/PKA) signaling pathway activates the cAMP response element-binding protein (CREB), that can translocate to mitochondria, inducing PGC-1α expression, and thus, mitogenesis.

In times of energy depletion, cells can sense low ATP levels via the serine/threonine kinase AMP-activated protein kinase (AMPK) complex, which induces mitogenesis via phosphorylation and activation of PGC-1α. Upregulation of mitogenesis regulatory proteins, especially PGC-1α, and a higher mitochondrial mass is associated with resistance to chemotherapy, as shown in studies with paclitaxel-treated breast cancer cells, selumetinib-resistant melanoma cell lines, and ovarian cancer cells treated with cisplatin.

- 1. Mitophagy

Mitochondria are degraded by a selective form of autophagy named mitophagy. This process occurs as a consequence of several stress and damage conditions including oxidative stress, hypoxia, mtDNA mutation, iron starvation, loss of mitochondrial membrane potential, and accumulation of unfolded proteins. Impairment of the mitophagy mechanism also correlates with different physiological processes, as differentiation, aging, and pathological disorders, like cancer and neurodegenerative diseases.

During mitophagy, mitochondria are embedded in double-membrane vesicles, known as autophagosomes, and degraded in lysosomes. Regulation of mitophagy is driven mainly by the E3 ubiquitin-ligase Parkin and the PTEN-induced kinase 1 (PINK1), ubiquitinating mitochondrial proteins and resulting in mitochondrial engulfment by lysosomes. Apart from the Parkin-related mitophagy pathway, several mitochondrial receptors can induce mitophagy, including the FUN14 domain containing protein 1 (FUNDC1), BCL-2-like protein 13 (BCL2L13), NIX (also called BCL2/adenovirus E1B-interacting protein 3-like, BNIP3L), and BNIP3. They all promote mitochondrial degradation by binding to the LC3B-II (Microtubule-associated protein 1A/1B-light chain 3) present in the mitochondrial autophagosomes.

- 1. Fusion-Fission

Mitochondrial dynamics are regulated by continuous fusion and fission processes, tightly related to cellular physiology and stress. The rapid morphological changes in mitochondria are critical for their function in different cellular processes including apoptosis, metabolism, immunity, and cell cycle. Fusion is enhanced when increased mitochondrial metabolism is needed, or to reduce stress in damaged mitochondria. Mitochondrial fission is the process in which one mitochondrion is divided in two. It is essential to ensure that dividing and growing cells have an adequate number of mitochondria, but also for apoptosis in situations of cellular stress and recycling damaged mitochondria (**Supplementary Figure 1**).

Both fusion and fission are controlled by guanosine triphosphatases (GTPases) from the dynamin family. In mammals, mitochondrial fusion is driven by the GTPases mitofusin 1 (MFN1) and mitofusin 2 (MFN2), both located in the OMM, and by the IMM-located optic atrophy 1 (OPA1) enzyme. Mitochondrial fusion is a two-step process that requires joining the OMM and IMM. When mitochondria are tethered by their OMM, GTP binding and hydrolysis changes MFN1 and MFN2 conformation increasing their contact sites and producing OMM fusion. During IMM fission, the interaction between OPA1 and cardiolipin, a specific lipid in the mitochondrial membrane, tethers the IMMs of both mitochondria. Consecutively, OPA1-dependent GTP hydrolysis results in IMM fusion.

Mitochondrial fission is carried out by the dynamin-related/-like protein 1 (DRP1) and dynamin 2 (DNM2), respectively (**Supplementary Figure 1**). This process starts with replication of mtDNA in the matrix, establishing the site for ER recruitment. DRP1 is recruited by fission 1 (FIS1) from the cytosol and anchored to the OMM, where the constriction starts at the mitochondria-ER contact sites. DRP1 oligomerization begins, forming a ring structure around the place of division. Hydrolysis of GTP leads to the conformational change of DRP1 and enhances mitochondrial constriction. Next, DNM2 is recruited resulting in two independent mitochondria. DRP1 activity is regulated by post-transcriptional modifications, especially phosphorylation, that affects its localization. Phosphorylation of DRP1 at Ser616, Ser537, and Ser600 initiates mitochondrial fission by promoting DRP1 translocation from the cytosol to the OMM, whereas phosphorylation at Ser637 and Ser656 reverses the process. Calcineurin, a cytosolic Ca^2+^-dependent phosphatase, dephosphorylates DRP1 at Ser656, promoting its translocation to the mitochondria.

**Supplementary Box 4**

**Mitochondrial Functions beyond Metabolism**

Although regulation of energy metabolism is the most known mitochondrial function, they are also involved in other processes including apoptosis, ROS and calcium signaling as well as iron metabolism.

- 1. Apoptosis

Apart from their important role in cellular metabolism, mitochondria are also implicated in the intrinsic apoptotic pathway. Upon death signals like DNA damage, growth factor deprivation, and an increased ROS levels, the mitochondrial membrane becomes permeable allowing the release of high molecular weight proteins including cytochrome c, second mitochondria-derived activator of caspase (Smac)/direct inhibitor of apoptosis-binding protein (Diablo), and endonuclease G. This process, known as mitochondrial outer membrane permeabilization (MOMP), occurs due to the oligomerization of BCL-2 Associated X (BAX)/BCL-2 antagonist killer (BAK) in the OMM. Next, cytochrome c forms a complex named apoptosome, by binding to the apoptotic peptidase activating factor 1 (APAF1), resulting in caspase 9 activation, cleavage and activation of caspase 7, and subsequently cell death.

- 1. ROS Signaling

The electron transport chain and the different enzymatic reactions occurring in mitochondria result in formation of superoxide radicals (O_2_^•–^), which in turn generate other ROS and reactive nitrogen species (RNS). Reactive oxygen and nitrogen species are not just metabolic by-products, instead they are second messengers controlling cell proliferation and survival. The amount of ROS/RNS determines the cellular outcome, as low levels sustain signaling pathways, but high levels induce oxidative stress. An imbalance between the production of reactive species and the antioxidant systems affects cancer initiation and progression. At the same time, oncogenes impact on ROS production and the expression and function of the antioxidant systems to maintain crucial signaling pathways while avoiding cellular death.

- 1. Mitochondrial-Nuclear Crosstalk

Mitochondria signal to the nuclei to regulate metabolic changes, which is known as “retrograde signaling”. This process is not limited to nuclear-encoded mitochondrial proteins, but to global nuclear transcription. Some of the pathways involved are nuclear factor of activated T-cells (NFAT), activating transcription factor 2 (ATF2) and 5 (ATF5), the latter involved in the activation of the mitochondrial unfolded protein response (UPR). In addition, nuclear factor kappa light chain enhancer of activated B cells (NF-κB), mitogen-activated protein kinase (MAPK), protein kinase C (PKC), and Cam-KIV-mediated activation of CREB. Moreover, mitochondrial metabolites can also serve as messengers for the nucleus. Fumarate, succinate, and 2-hydroxyglutarate, act as epigenetic regulators controlling DNA methylation, and acetyl-CoA influences histone acetylation, thus affecting cellular transcription.

**Supplementary Box 5**

**Other Mutations in TCA Enzymes in Cancer**

Citrate synthase (CS) is the rate limiting enzyme of the TCA cycle, where oxalacetate and acetyl-CoA are converted to citrate. Citrate synthase knockout (KO) results in a metabolic shift from OXPHOS to glycolysis and EMT in different cancer cell lines associated with aggressiveness. The enzyme is upregulated in pancreatic, prostate, and ovarian cancer. Reducing *CS* expression in ovarian cancer cells lines led to decreased proliferation and migration.

Aconitase 2 (ACO2) is responsible for the reversible isomerization of citrate to isocitrate. The cytoplasmic version of this reaction is performed by aconitase 1 (ACO1). The *ACO2* gene is inactivated in *FH*-deficient tumors and the levels are decreased in several cancer types. For instance, overexpression of the enzyme in breast cancer cells results in inhibition of proliferation and a shift to oxidative metabolism. In colorectal cancer cells, *ACO2* KO promotes cell proliferation while its inhibition reduces oxidative phosphorylation, increasing glycolysis and citrate usage for lipid synthesis, favoring tumor growth.

The multienzyme α-KG dehydrogenase complex (KGDHC) catalyzes the conversion of α-KG to succinyl-CoA and includes three components: α-KG dehydrogenase (OGDH), dihydrolipoamide S-succinyltransferase (DLST), and dihydrolipoamide dehydrogenase (DLD). α-KG dehydrogenase (OGDH) is upregulated and contributes to gastric cancer progression by increasing mitochondrial function, activating Wnt/β-catenin signaling and the expression of EMT transcription factors. Moreover, MYC-driven T-cell acute lymphoblastic leukemias depend on the DLST component of KGDHC for cell growth and survival. In addition, a high dependency on OGDH in several cancer cell types has been reported.

The conversion of pyruvate into oxalacetate by pyruvate carboxylase (PC) is one of the major anaplerotic reactions. This enzyme is also important for lipogenesis and generation of reducing equivalents, protecting from oxidative stress. Due to its central role, PC regulation is important for metabolic plasticity in cells. Its expression is upregulated in different types of cancer, including breast, lung, gallbladder, and papillary thyroid cancer, and it is necessary for breast cancer cells growth after dissemination to the lungs. In non-small cell lung cancer (NSCLC), *PC* KO cells decreased cell proliferation and colony formation *in vitro* and reduced tumor burden *in vivo*.

**Supplementary Box 6**

**TCA Cycle Derived Oncometabolites**

- 1. 2-Hydroxyglutarate

*IDH1* and *IDH2* mutations confer cancer cells the capacity to reduce α-KG to 2-hydroxyglutarate (2HG). Due to its chiral carbon-2, it has two enantiomers: D- and L-2HG. While D-2HG accumulates in leukemias and gliomas, L-2HG is elevated in ccRCC. Under hypoxic conditions in renal cancer, L-2HG is synthetized by LDHA and malate dehydrogenase (MDH). 2-hydroxyglutarate (2HG) inhibits α-KG dependent dioxygenases, including jumonji-C (JmjC) histone demethylases and the TET family of 5-methlycytosine (5mC) hydroxylases, causing alterations in histone and DNA methylation. Restoring the activity of TET2 in ccRCC inhibits proliferation and tumor growth. Moreover, treatment with 2-HG results in decreased T cell-attracting chemokines and suppressed cytotoxic T cell accumulation in gliomas and activation of NF-κB gene transcription in stromal cells promoting a tumor niche for acute myeloid leukemia. Furthermore, MYC activation in aggressive breast tumors results in an increase of 2-HG leading to DNA hypermethylation similar to tumors with *IDH* mutations.

- 1. Succinate

Succinyl-CoA is formed by oxidative decarboxylation of α-KG by α-KG dehydrogenase, and is the precursor of succinate. Mutations in *SDH* results in accumulation of both succinate and succinyl-CoA. Histone succinylation by succinyl-CoA modulates gene expression and impairs DNA repair, suggesting a possible effect on cancer progression. Equally to 2-HG, succinate and fumarate are also inhibitors of α-KG-dependent dioxygenases. In normoxia, prolyl-hydroxylases (PHDs), from the family of α-KG-dependent dioxygenases, mediate the hydroxylation of two proline residues in the HIF-1/2α. The hydroxylation sites are recognized by the VHL (von Hippel-Lindau) tumor suppressor, targeting it for proteasomal degradation. During hypoxia or in cells lacking *VHL*, the HIF1/2-α protein is stabilized and dimerizes with HIF-1β. The complex activates expression of the hypoxia response genes, increasing angiogenesis and glycolysis, both cancer-associated processes. Accumulation of succinate creates a pseudohypoxic environment by inhibiting the activity of PHDs that can be reverted by an increase of α-KG levels. Moreover, it has been demonstrated that the secreted succinate by tumor cells promotes polarization of tumor associated macrophages (TAMs) and induces migration of cancer cells via PI3K/HIF-1α pathway.

- 1. Fumarate

Hydration of fumarate to malate by fumarate hydratase prevents its accumulation in cells. Fumarate can also be produced by the urea and purine nucleotide cycles. High levels of this oncometabolite are also associated with cancer. Inhibition of α-KG-dependent oxygenases, specially PHDs, creates a pseudohypoxic environment stabilizing HIF levels and activating hypoxia response genes, associated to oncogenesis. Moreover, fumarate also inhibits the action of TET demethylases, suppressing *miR-200* and promoting EMT associated with cancer migration and metastasis. In addition, fumarate is an endogenous electrophile that reacts with cysteine residues producing *S*-(2-succinyl) cysteine (2SC), a process termed succination. An increase in 2SC has been observed in *FH-*deficient tumors compared to normal tissues. Succination of the kelch-like ECH-associated protein 1 (KEAP1) upon fumarate accumulation results in stabilization of NRF2 (nuclear factor erythroid 2- related factor 2) activating expression of genes that help tolerating high ROS levels in cancer cells.

**Supplementary Box 7**

**Electron Transport Chain (ETC)**

﻿The electron transport chain consists of five different complexes. Complex I (NADH-Ubiquinone Oxidoreductase) includes NADH dehydrogenase, flavin mononucleotide (FMN), iron-sulfur (Fe-S) clusters, and a final electron accepting Fe-S cluster (N2). When NADH generated during glycolysis and the TCA cycle is oxidized, two electrons flow to FMN, then to the Fe-S clusters and finally, ubiquinone (coenzyme Q, CoQ) is reduced to ubiquinol (CoQH2). Every NADH molecule results in the translocation of four protons. The second entry point to the ETC is complex II (Succinate Dehydrogenase). FAD accepts two electrons from the reduction of succinate to fumarate and electrons pass from FADH2 to three Fe-S clusters and CoQ. The released energy in this complex is not enough to pump protons. Once electrons from complexes I and II pass to CoQ, it undergoes reduction to semi-ubiquinone (CoQH^-^) and CoQH2. Complex III (Ubiquinol-Cytochrome c Oxidoreductase) consists of cytochrome b (bL and bH), cytochrome c1 and a 2Fe-2S cluster wrapped by an iron-sulphur protein. The transfer of electrons from ubiquinol (QH_2_) to complex III takes place through the Q-cycle. Every electron is transferred at a time and the energy after a full Q-cycle (2 electrons) results in the pumping of four protons. Complex IV (Cytochrome c Oxidase) oxidizes cytochrome c and transfers electrons, one at a time, to oxygen that converts to water molecules. This complex consists of thirteen different subunits that are responsible for the reduction of O_2_ to H_2_O. The two-electron transfer causes four protons to move into the intermembrane space. Finally, complex V (F_1_F_0_ATP synthase) uses the ETC proton gradient across the inner mitochondrial membrane to form ATP, one every four H^+^. Oxidative phosphorylation is not completely coupled, and some protons can flow into the mitochondrial matrix instead of passing through the ATP synthase, the so called proton leak.

**Supplementary Box 8**

**Fatty Acid β-Oxidation (FAO)**

Free fatty acids are unable to move through biological membranes. To enter mitochondria, they are initially activated in the cytosol by binding to Coenzyme A (CoA) in an ATP-consuming reaction producing fatty acyl-CoA. The enzyme carnitine palmitoyltransferase 1 (CPT1), the rate-limiting enzyme of FAO, catalyzes fatty acyl to a carnitine, releasing CoA at the cytosolic side of the OMM. The resulting fatty acyl-carnitine is transferred to the mitochondrial matrix by the carnitine-acyl carnitine translocase, moving one molecule of free carnitine to the intermembrane space at the same time. Once in the matrix, CPT2 catalyzes the opposite reaction previously performed by CPT1, binding the fatty acyl to CoA, and releasing carnitine. The fatty acyl CoA is then degraded by the cyclic reactions of FAO, including the following enzymatic activities: acyl-CoA dehydrogenase, enoyl-CoA hydratase, 3-hydroxyacyl-CoA dehydrogenase, and acyl-CoA acetyltransferase (or thiolase). Each cycle results in the shortening of fatty acids by two carbon atoms, released in the form of acetyl-CoA which can be further oxidized through the TCA cycle, and for production of one molecule of NADH and one of FADH_2_, which in turn will donate electrons to the ETC for ATP production. The final cycle will break down one four carbon-long fatty acid into two molecules of acetyl-CoA (please refer to **Figure 4**). Short fatty acids (less than six carbons) can diffuse through the IMM while very long chain ones (with 22 carbons or more) are not processed by mitochondrial FAO but in the peroxisomes.

**Supplementary Box 9**

**Glutamine Metabolism in Mitochondria**

In many cases, glutamine is the main TCA substrate in rapidly proliferating malignant cells. Its importance for cancer proliferation was first described by Eagle, demonstrating that HeLa cells require 10-to-100-fold molar excess of glutamine for optimal growth. Oncogenes such as *RAS* and *MYC* are responsible for this metabolic adaptation. To divert glutamine-derived carbons into the TCA cycle, mitochondrial glutaminases (GLS and GLS2), convert glutamine into glutamate. Glutamate dehydrogenase (GDH) subsequently converts glutamate to α-ketoglutarate. Alternatively, glutamate can be trans-aminated by alanine aminotransferase (GPT2) or aspartate aminotransferase (GOT2) to produce alanine and α-ketoglutarate or aspartate and α-ketoglutarate, respectively. α-ketoglutarate can enter the TCA cycle in a “backwards” flux called reductive carboxylation, getting reduced by IDH in a non-canonical reverse reaction to produce citrate. Citrate can in turn be shuttled to the cytosol for synthesis of fatty acids. Together with its role as an anaplerotic source, glutamine-derived glutamate can also be used in mitochondria to synthesize glutathione, serine, glycine (also constituents of glutathione), or proline. In addition, glutamine serves as a nitrogen donor for both *de novo* pyrimidine and purine synthesis. While purine synthesis occurs in the cytosol, pyrimidine synthesis is compartmentalized between cytosol and mitochondria, with the synthesis of orotate by dihydroorotate dehydrogenase (DHODH) occurring in the IMM.

**Supplementary Box 10**

**Reverse Electron Transport**

In some situations, the electron chain acts in reverse (RET), and electrons are transferred from UbQH_2_ back to complex I, with the generation of NADH from NAD^+^ and significant amounts of ROS. This process depends on the proton motive force (Δp), which indicates the ATP molecules mitochondria can produce, and the redox state of the CoQ pool, which is determined by several metabolic pathways. Indeed, RET-ROS serve as communication system from the mitochondria to other parts of the cell. It has roles both in physiological conditions (myoblast differentiation, macrophage functioning, anti-aging) and pathological (ischemia-reperfusion injury).

**Supplementary Box 11**

**Mitochondrial ROS**

Superoxide is produced at different sites of the respiratory chain, mainly in complex I (at sites I_Q_ and I_F_) and complex III (at site III_Qo_), but also in complex II (at site II_F_). Complex IV is indirectly involved in ROS production, as the overall electron flow depends on its activity. Mitochondrial enzymes can also produce superoxide as the result of the electron leakage during their reactions. For instance, proline dehydrogenase (PRODH), glycerophosphate dehydrogenase (GPDH/GPD2), mitochondrial DHODH, mono-amino oxidase (MAO), 2-oxoglutarate dehydrogenase (OGDH), and pyruvate dehydrogenase (PDH).

Chronic lymphocytic leukemia (CLL), acute myeloid leukemia (AML), paraganglioma, PCC, RCC, and gastrointestinal stromal tumor (GIST) are examples of tumors with high mtROS. In addition, mutations in the ETC found in multiple tumors lead to the impairment of the electron transfer and formation of mtROS. In melanoma, cells at metastatic lesions contained more mtROS than cells in circulation or in the primary tumor. Moreover, some subsets of cancer stem cells (CSCs) contain lower mtROS levels and higher antioxidant systems compared to non-tumorigenic cells, what results in less DNA damage and less sensitivity to irradiation.

**Supplementary Box 12**

**Metabolism of Ketone Bodies**

Ketogenesis takes place after fatty acids are transported into mitochondria and catabolized into acetyl-CoA by β-oxidation. The enzyme 3-hydroxymethylglutaryl-CoA synthase (HMGCS2) generates hydroxymethylglutaryl (HMG-CoA) from acetoacetyl-CoA (AcAc-CoA) and acetyl-CoA. Next, hydroxymethylglutaryl-CoA lyase (HMGCL) cleaves HMG-CoA resulting in acetyl-CoA and acetoacetate (AcAc). Acetoacetate is reduced to D-β-hydroxybutyrate (D-βOHB) by phosphatidylcholine-dependent mitochondrial D-βOHB dehydrogenase (BDH1). The activity of this enzyme is coupled to NAD^+^/NADH, thus, modulating mitochondrial redox potential. Acetone is produced by spontaneous decarboxylation of acetoacetate, and then excreted in the urine or exhaled from the lungs. Acetoacetate and D-βOHB are released from hepatocytes through monocarboxylate transporters (MCT1 and MCT2) and used in extrahepatic tissues, either for catabolism or for lipogenesis or sterol synthesis. First, BDH1 converts βOHB to acetoacetate, and then succinyl-CoA-ketoacid-CoA transferase (SCOT) adds a CoA, resulting in acetoacetyl-CoA (AcAc-CoA). Finally, mitochondrial acetyl-CoA thiolases produce two molecules of acetyl-CoA, which enter the TCA cycle. Ketones are considered a super fuel, as they provide more acetyl-CoA molecules than glucose.

Ketogenic diet has been widely used for the treatment of epilepsy due to its anti-seizure effect (reviewed in Hartman and Vining, 2007). This high-fat diet prevents glutamate excitotoxicity by reducing ROS levels. The antioxidant effect of ketones has been explained by different mechanisms. An early increase in ketone bodies generates more ROS, which in turn activates the NRF2 transcription factor. In turn, NRF2 induces expression of the antioxidant response genes, which reduces ROS levels. Ketone bodies also increase the NAD^+^/NADH ratio, improving the mitochondrial redox status and protecting against oxidative stress. They rise the mitochondrial uncoupling protein activity (UCP), resulting in a lower membrane potential and reducing the production of superoxide. Ketogenic diet also stimulates mitochondria biogenesis and respiration rates and it has been associated with low insulin levels, a decrease in histone deacetylase activity (HDAC), and mTOR as well increased FOXO3.
